# Supplementary material for: Using language in social media posts to study the network dynamics of depression longitudinally
Source: Nat Commun. 2022 Feb 15;13:870. doi: 10.1038/s41467-022-28513-3 (PMC8847554; doi:10.1038/s41467-022-28513-3)
Supplement: Supplementary file 1 — Supplementary Information [file 41467_2022_28513_MOESM1_ESM.pdf]

Supplementary Material

CHARACTERISTICS OF PARTICIPANTS RECRUITED THROUGH PAID VS FREE CHANNELS 2

RELIABILITY OF ESTIMATED EDGES IN 9-NODE NETWORK 3

SENSITIVITY OF OUR ANALYSIS TO THE REMOVAL OF 'SUPRA' CATEGORIES IN LIWC 5

THE EFFECT OF NUMBER OF DAYS ON GLOBAL NETWORK CONNECTIVITY AND ASSOCIATED ANALYSES 7

FREQUENCY OF OCCURRENCES OF THE 9 *A PRIORI* TEXT FEATURES IN THE 12-MONTH TIME SERIES 8

ASSOCIATION BETWEEN NETWORK CONNECTIVITY AND DEPRESSION SEVERITY IS NOT AFFECTED BY NUMBER OF DAYS 9

THE ASSOCIATION BETWEEN NETWORK CONNECTIVITY AND DEPRESSION SEVERITY IS NOT AFFECTED BY REMOVAL OF 3RD PERSON PRONOUNS 10

BOOTSTRAPPED CONTROL FOR UNEQUAL VARIANCES IN WITHIN-EPISODE NETWORK CONNECTIVITY 11

PERMUTATION TEST OF WITHIN-EPISODE IDENTIFER 12

NO MEAN INCREASE IN THE USE OF 9 *A PRIORI* TEXT FEATURES OUTSIDE VS WITHIN A DEPRESSIVE EPISODE 14

ASSOCIATION BETWEEN ALL LIWC TEXT FEATURES AND CURRENT DEPRESSION SEVERITY 15

Characteristics of participants recruited through paid vs free channels

Table S1. Demographics and Twitter use characteristics of subjects recruited through paid (ClickWorker) vs free channels

	Paid<br>N = 680	Free<br>N = 266	p-value
Twitter Behaviour			
Tweets	294.8 (537.5)	512.6 (656.4)	1.9e-07***
Retweets	230.5 (473.8)	392.5 (525.7)	5.3e-06***
Likes	1033.9 (1090.3)	1260.9 (971.6)	0.003
Word count per day	117.2 (121.8)	158.1 (139.0)	1.2e-05***
Age (years)	29.6 (10.0)	29.5 (12.1)	0.92
Gender			
Male	216 (31.8%)	88 (33.1%)	0.77[a]
Female	443 (65.1%)	174 (65.4%)	-
Transgender Male	5 (0.7%)	1 (0.4%)	-
Transgender Female	1 (0.1%)	0 (0%)	-
Non-Binary	13 (1.9%)	2 (0.8%)	-
Other	2 (0.3%)	1 (0.4%)	-
Country			
Ireland	19 (2.8%)	25 (9.4%)	3.2e-17[a]***
United Kingdom	281(41.3%)	58 (21.8%)	-
United States	334 (49.1%)	146 (54.9%)	-
Canada	32 (4.7%)	10 (3.8%)	-
Australia	11 (1.6%)	4 (1.5%)	-
Other	3 (0.4%)	23 (8.6%)	-
Education			
Less than high school	11 (1.6%)	5 (1.9%)	5.5e-06[a]***
High school	154 (22.6%)	36 (13.5%)	-
Some university	210 (30.9%)	107 (40.2%)	-
Bachelor's degree	221 (32.5%)	64 (24.1%)	-
Master's degree	70 (10.3%)	34 (12.8%)	-
Professional degree	6 (0.9%)	9 (3.4%)	-
Doctorate	8 (1.2%)	11 (4.1%)	
Currently Employed (% Yes)	436 (64.1%)	22 (8.3%)	2.2e-53[a]***
Physician diagnosed depression (% Yes)	292 (42.9%)	140 (52.6%)	0.01[a]*

Twitter and demographic characteristics of all participants along with differences between participants with and without a depressive episode. [a]Chi-square test. Source data are provided as a Source Data file.

*p < 0.05, **p < 0.01, ***p < 0.001

Reliability checks on a 9-node network

We conducted a split half reliability test to determine if the 36 unique edges in our network were reliable. We did this across subjects by splitting our sample into two equal halves, calculated personalised networks for all participants, and then correlated the mean edge strengths between the two halves. We found the estimates of edge strength to be highly reliable r(36)=.99, p<.001. The edge strength between Neg. Emo. and swear was much stronger than between other edges, reliability was r(35) = 0.97, p < 0.001 when we exclude this edge. Because individual networks tended to be sparse, the average of most edges tended towards zero leading to a high correlation between split halves.

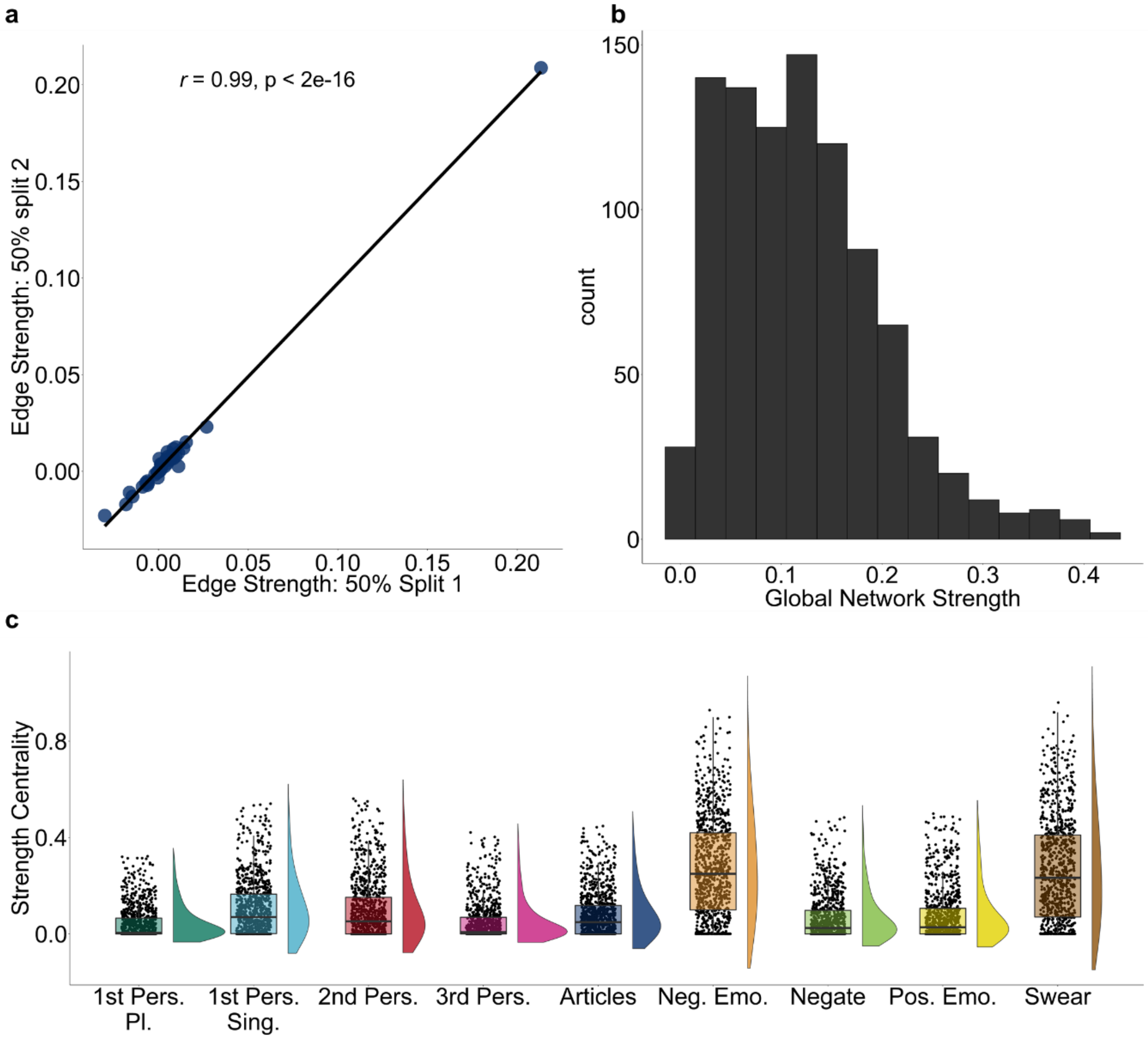

Figure S1. Split half reliability of primary 9-node network. a): The edge strength among 36 unique connections between nodes in split halves (n = 473 for each half) of the sample were highly correlated with each other (r(36) = 0.99, p < 2e-16), indicating that the estimated edge strengths between nodes are highly reliable. b): Histogram of global network strength from personalised networks of all participants (n = 946). c) Variability of node strength centrality in the 9-node network (n = 946). Boxplots depict the median (centre line), upper and lower quartiles, i.e., interquartile range, whiskers, 1.5X interquartile range, and minimum and maximum values. [a]Unadjusted two-sided Pearson correlation. Source data are provided as a Source Data file.

Sensitivity of our analysis to the removal of ‘Supra’ categories in LIWC

In the LIWC library, certain categories are inclusive of multiple sub-categories. For example, within the personal pronoun category are: 1st person singular/plural, 2nd person, 3rd person, and impersonal pronouns. An increase in 1st person singular pronouns will then necessarily lead to an increase in the proportion of pronouns overall. To control for this potential, confound, the following supra-categories were removed from inclusion in the network: function words, impersonal pronouns, pronouns, affect, anxiety, anger, sad, social, cognitive processes, percept, biological, drives, relative, and informal. We then randomly selected 200 sets of 9 text features that were significantly (‘Depression Relevant’ x 100) or not-significantly (‘Depression Irrelevant’ x 100) associated with current depression severity. We then compared the change in within-episode connectivity between depression relevant and irrelevant networks. There was a significant increase in within-episode connectivity (β = 0.01, SE = 0.001, p < 0.001) even after removing the supra-categories within the LIWC. Thus, the increase in within-episode connectivity for depression relevant networks is not dependent on the inclusion of LIWC supra-categories.

Figure S2: Random networks of 9 text features either significantly (‘Depression Relevant’) or not significantly (‘Depression Irrelevant’) associated with current depression excluding LIWC supra-categories.

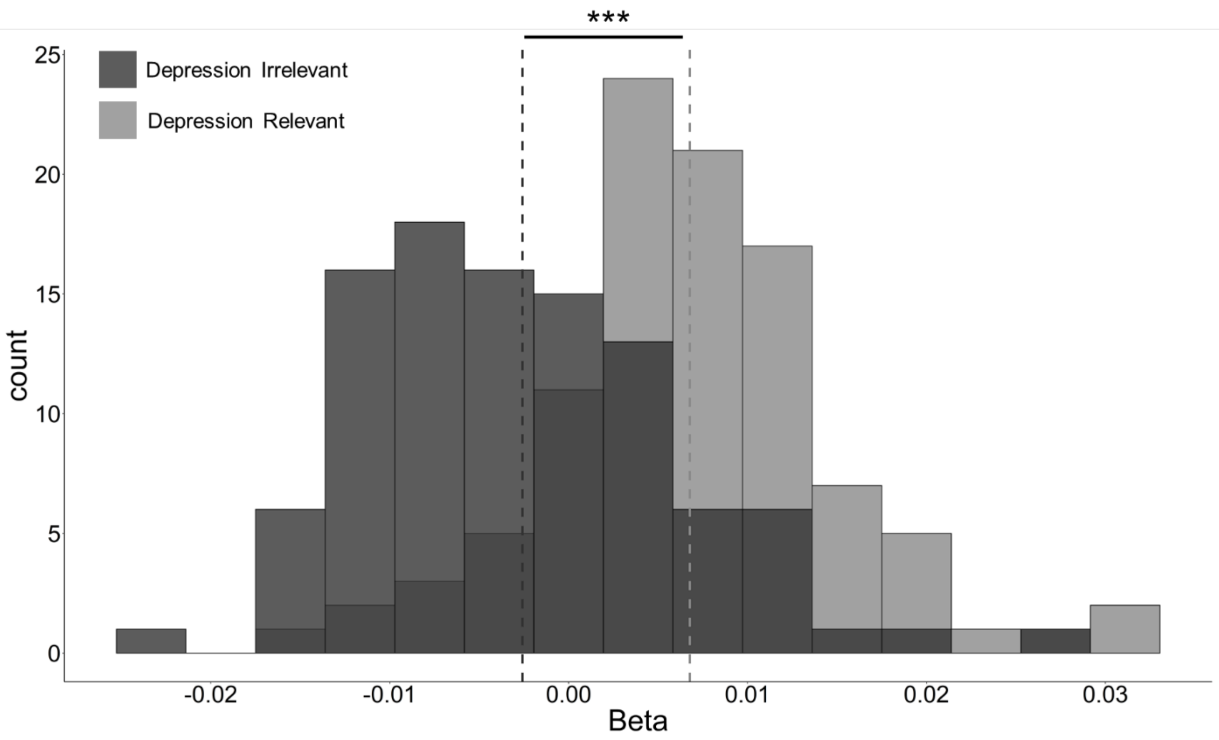

There was a significant increase in within-episode network connectivity in depression relevant vs. depression irrelevant networks ($\beta = 0.01$, SE = 0.001, p = 2.4e-13). Results are from a general linear model with two-sided p-values without adjustment for multiple comparisons. Source data are provided as a Source Data file.

*p < 0.05, **p < 0.01, ***p < 0.001

The effect of number of days on global network connectivity and associated analyses

We were concerned that between and within-subject differences in the number of days upon which we based our 9-node a priori network might affect the connectivity results. Indeed, we observed that subjects with more days had on average, more connected networks, $r=.19$, $p<.001$ (Figure S1a). Next, we checked if this might systemically bias our between-subject findings by testing for an association with depression symptom severity and the number of days with tweets. We found no association (Figure S1b). However, we found a significant within subject effect such that periods of time when subjects were in a depressed episode were significantly shorter than non-depressed periods of time (Figure S1c), β = -90.8, SE = 6.2, $p < 0.001$. We examined the network connectivity of within and between episode time periods (Figure S1d) and found a significant interaction (β = 0.0005, SE = 0.0001, $p < 0.001$) between the number of days and episode (within vs. outside).

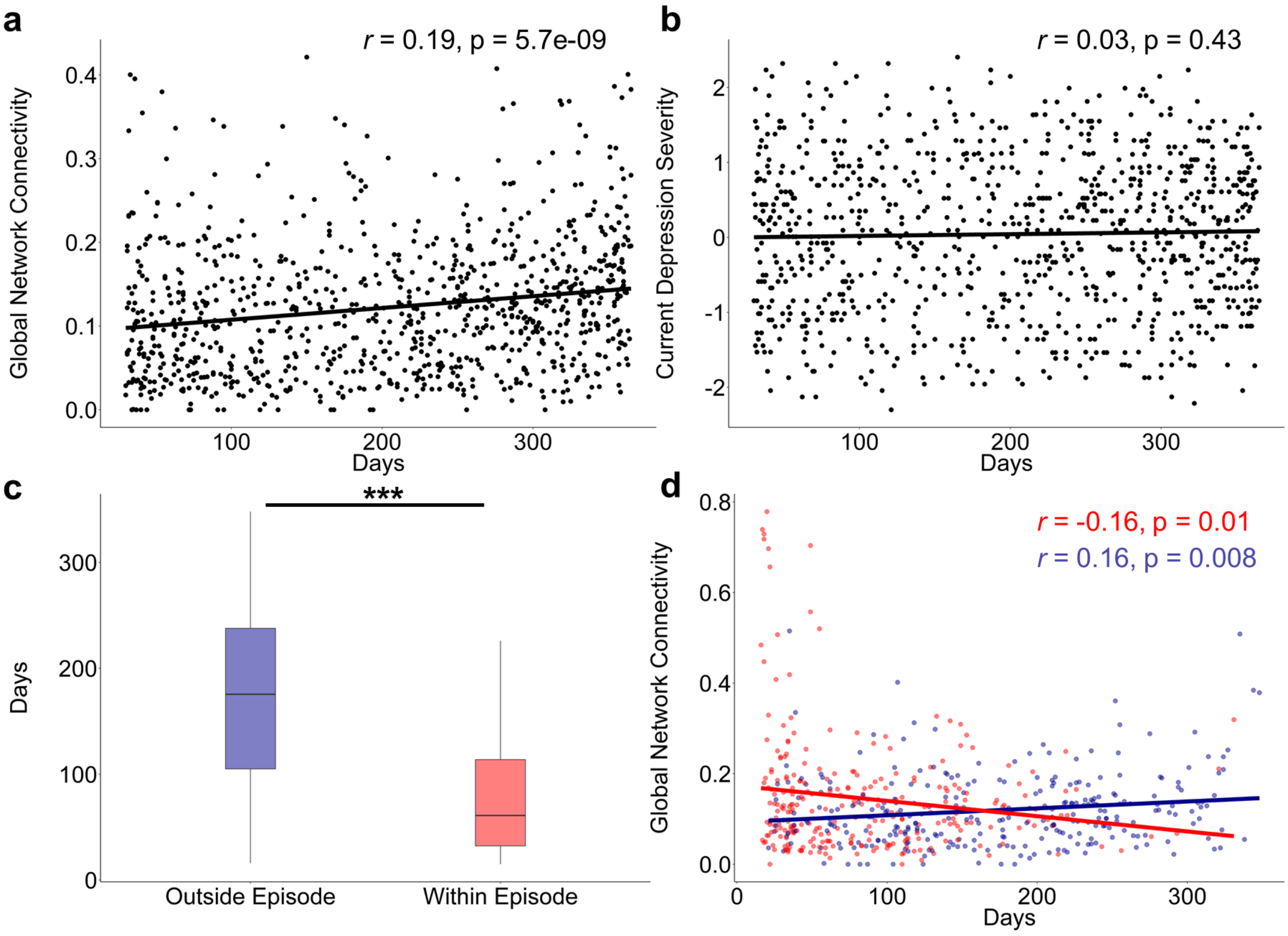

Figure S3. The effect of days on global network connectivity.

a) Among all participants (N = 946), there was a significant positive association between global network connectivity and number of days (r(936) = 0.19, p = 5.7e-09) in the full sample b).There was no significant association between current depression severity and number of days (r(944) = 0.03, p = 0.43). c) Within-episode periods have significantly fewer days on average than outside episode periods (β = -90.8, SE = 6.2, p < 2e-16). d) Association between number of days and within vs. outside episode network connectivity. There is significant interaction effect between the number of days and whether data is from a depressed vs non-depressed episode (β = 0.0005, SE = 0.0001, p < 0.001). For non-depressed periods of time (blue), the more days there were, the greater connectivity the connectivity, but for depressed episodes, the relationship was reversed. The boxplot depicts the median (centre line), upper and lower quartiles, i.e., interquartile range, and whiskers, i.e., 1.5x interquartile range. [a,b,d]Unadjusted two-sided Pearson correlation, [c,d]Two-sided general linear regression model. Source data are provided as a Source Data file.

As there was a credible confound from differences in days within subject, to control for the effect of the number of days on connectivity estimates, we repeated our analysis including days as a covariate (Table S2). This did not alter the significant positive association between current depression severity and negative emotions, swear words, articles, and global network connectivity in the full sample of participants (N = 946).

Frequency of occurrences of the 9 a priori text features in the 12-month time series

For each subject, we calculated the proportion of their days with tweets that contained each of the 9 text features. Swear was the least frequent, appearing on average in 30% of days with tweets, but with some individuals never swearing and others swearing on 100% of days with tweets. Articles were the most frequent, appearing on average in 80% of days with tweets, with the lower bound being 22% and upper 100%.

Table S2. Proportion of days with non-zero values for each of the 9 a priori text features

Text Feature	Proportion of Days with Non-Zero Text Features (Mean, SD) Range
1st Person plural	0.38 (0.21) [0.02,1]
1st Person singular pronouns	0.69 (0.20) [0.08,1]
2nd Person singular pronouns	0.57 (0.20) [0.04,1]
3rd Person pronouns	0.50 (0.22) [0.02,1]
Articles	0.80 (0.14) [0.22,1]
Negate	0.55 (0.22) [0.03,1]
Negative emotions	0.60 (0.22) [0.07,1]
Positive emotions	0.75 (0.16) [0.24,1]
Swear	0.30 (0.22) [0.00,1]

Table S3: Controlling for days with tweets: adjusted vs unadjusted analyses for the association between depression severity and network connectivity

LIWC Text Feature	Unadjusted β	SE	p-value	Adjusted β	SE	p-value
Global Network Connectivity	0.008	0.003	0.002**	0.007	0.003	0.004**
Articles	0.01	0.003	<0.001***	0.01	0.003	<0.001***
1st person singular pronouns	0.007	0.004	0.07	0.006	0.004	0.09
Negation words	0.003	0.003	0.36	0.003	0.003	0.36
Negative emotions	0.002	0.007	0.007**	0.02	0.007	0.009**
Positive emotions	0.006	0.004	0.12	0.006	0.004	0.12
3rd person pronouns	0.005	0.003	0.08	0.005	0.003	0.08
Swear words	0.02	0.007	0.01*	0.02	0.007	0.01*
1st person plural pronouns	0.0005	0.002	0.82	0.0004	0.002	0.85
2nd person singular pronouns	0.006	0.004	0.12	0.006	0.004	0.13

The association between network connectivity and depression severity is not affected by number of days (n = 286). Unadjusted within-subject linear model. Source data are provided as a Source Data file.

The association between network connectivity and depression severity is not affected by removal of 3rd person pronouns

Unlike the 8 other nodes selected a priori, there was no cross-sectional association with current depression severity and 3rd person pronouns. We therefore conducted a sensitivity analysis to test for the effect of removing 3rd person pronouns, she/he and they, on network structure. There was a significant positive association between overall network connectivity and current depression severity (β = 0.008, SE = 0.003, p = 0.003). Along with a significant increase in the node strength of articles (Article, β = 0.01, SE = 0.003, p < 0.001), swear words (Swear, β = 0.02, SE = 0.007, p = 0.02), and negative emotions (Neg. Emo., β = 0.02, SE = 0.007, p = 0.02). We found no significant changes to associations between network structure and depression severity caused by removing 3rd person pronouns.

Figure S4. Tolerance of within subject analysis to the removal of 3rd person pronouns

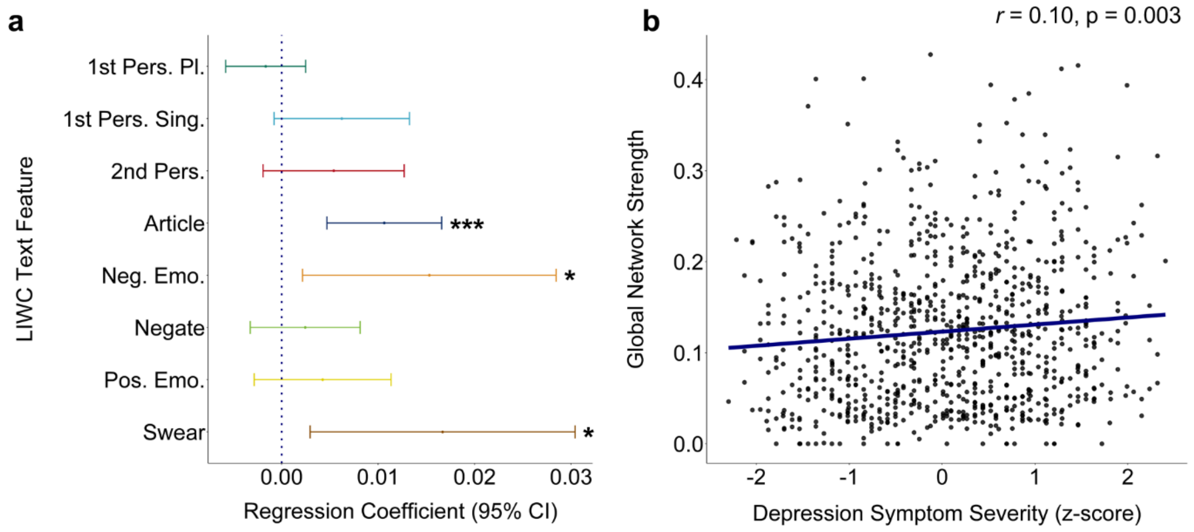

Sensitivity analysis to test for the effect of removing 3rd person pronouns, she/he and they, on network structure due to lack of association with current depression severity. a) The association between individual network node strength and depression symptom severity with significant associations for the node strength of articles (Article, β = 0.01, SE = 0.003, p = 0.0005), swear words (Swear, β = 0.02, SE = 0.007, p = 0.02), and

negative emotions (*Neg. Emo.*, $\beta = 0.02$, SE = 0.007, p = 0.02) (n = 286). b) The association between global network connectivity for personalised networks of all participants (N = 946) and depression symptom severity. [a]Unadjusted within-subject linear regression, [b]Two-sided Pearson correlation unadjusted for multiple comparisons. Source data are provided as a Source Data file.

*p < 0.05, **p < 0.01, ***p < 0.001

Bootstrapped control for unequal variances in within-episode network connectivity

To control for the unequal variances in the distribution of within vs. outside episode network connectivity, we subsampled 80% of global network connectivity from personalised networks of participants within and outside a depressive episode and re-ran the within-subject regression 1,000 times. We found that after bootstrapping the within-episode regression coefficient that the change in within-episode connectivity was still significant ($\beta = 0.03$, SE = 0.0001, p < 0.001).

Figure S5: Bootstrapped regression coefficient of change in network connectivity within a depressive episode from 80% random sub-samples of the data repeated 1,000 times

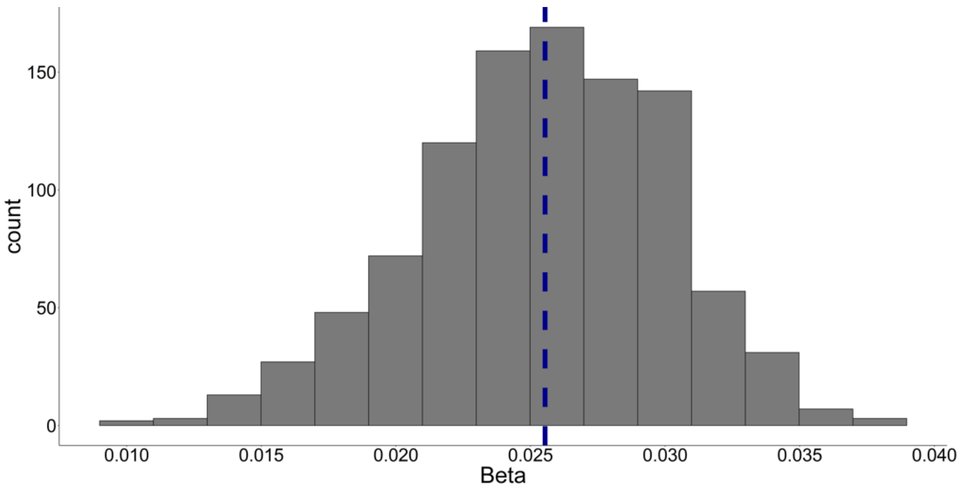

Permutation test of within-episode identifier

We conducted a permutation test, randomising the indicator (within/outside episode) per subject 1000 times and comparing network strength within subject using this indicator. This amounts to comparing network connectivity for a random period of time (that has the same number of days as the real within episode period) to another random period of time (that has the same number of days as the real outside episode period). Networks were constructed using the same 9 LIWC text features as used in the main analysis and positive betas indicate that the fake 'within episode" network had more connectivity than the fake 'outside episode' one. These figures show that (A) 99.3% of betas were smaller than the true within/outside episode value in the unshuffled data, and (B) 11% of p-values were below 0.05. This indicates the discrepancy in number of days had an influence on global network connectivity differences, but this does not explain the findings within and outside episode.

Figure S6. Permutation test randomizing within versus outside episode indicators.

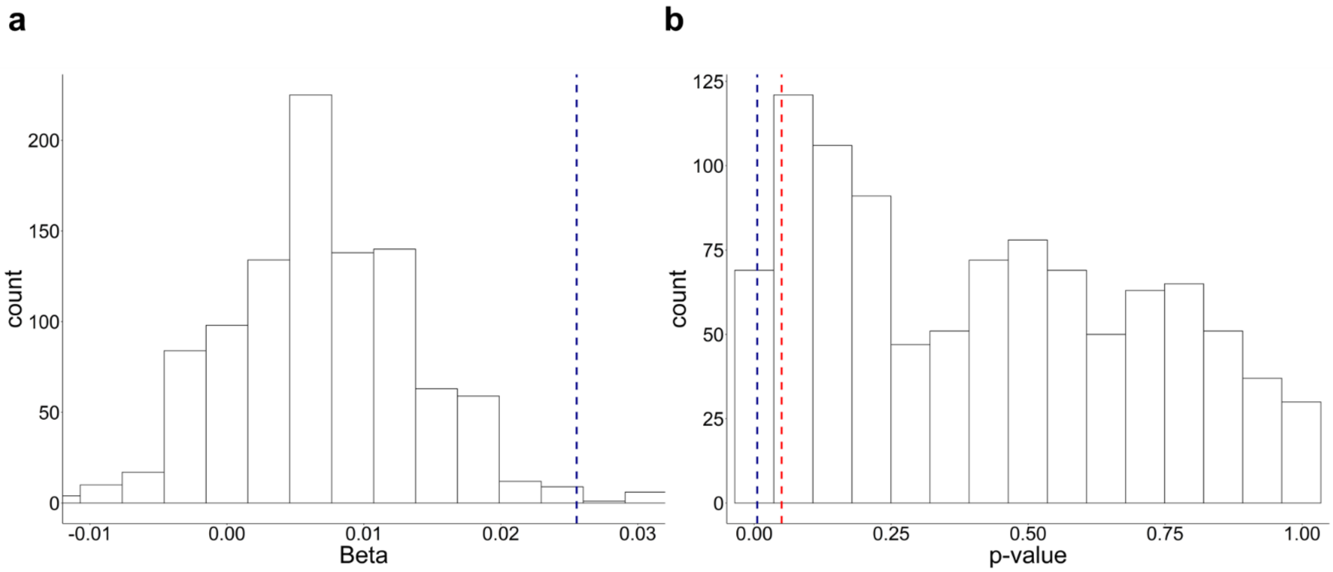

Blue dashed lines indicate the observed beta and observed p-value for our a priori depression network, the red dashed line indicates the alpha level of 0.05. These figures show that (a) 99.3% of betas were smaller than the true within/outside episode value in the unshuffled data, and (b) 11% of p-values were below 0.05. [a]Number of regression coefficients from 1,000 within-subject linear models that the observed beta value is

below, unadjusted two-sided p-value, [b]Number of p-values from the same 1,000 within-subject linear models that the observed p-value is below. Source data are provided as a Source Data file.

No mean increase in the use of 9 *a priori* text features outside vs within a depressive episode

In contrast to our principal findings that network connectivity increases when subjects are in a depressive episode, we found no significant change in any LIWC text feature within a depressive episode ($p > 0.05$).

Table S4. Results from regression analysis of the effect of episode (within, outside) on the mean use of the 9 a priori text features (N=286)

LIWC Text Feature	β	SE	p-value
1st person plural pronouns	-0.02	0.01	0.12
1st person singular pronouns	-0.05	0.04	0.31
2nd person singular pronouns	-0.03	0.03	0.32
3rd person pronouns	0.01	0.01	0.38
Articles	-0.03	0.04	0.48
Negation words	-0.03	0.02	0.14
Negative emotions	-0.01	0.03	0.76
Positive emotions	-0.02	0.04	0.63
Swear words	0.01	0.01	0.69

Mean difference in LIWC text features within a depressive episode compared to outside an episode from a within-subject regression (N = 286). Two-sided general linear regression unadjusted for multiple comparisons. Source data are provided as a Source Data file.

Association between all LIWC text features and current depression severity

Table S5: Bivariate correlations for all LIWC features and depression severity in full sample

LIWC Text Feature	*r*	95% CI	p-value
Conjunction	0.17	0.1, 0.23	< 0.001***
Negative emotions	0.17	0.11, 0.23	< 0.001***
Biological	0.17	0.1, 0.23	< 0.001***
Adverb	0.16	0.09, 0.22	< 0.001***
Sad	0.16	0.09, 0.22	< 0.001***
Analytic	-0.15	-0.21, -0.09	< 0.001***
Verb	0.15	0.09, 0.21	< 0.001***
Anxiety	0.15	0.08, 0.21	< 0.001***
Anger	0.15	0.08, 0.21	< 0.001***
Focus on present	0.15	0.09, 0.21	< 0.001***
Word Count	0.14	0.07, 0.2	< 0.001***
I (1st person singular)	0.14	0.08, 0.20	< 0.001***
Sexual	0.14	0.07, 0.2	< 0.001***
Pronouns	0.13	0.07, 0.19	< 0.001***
Space	-0.13	-0.19, -0.07	< 0.001***
Words per sentence	0.12	0.05, 0.18	< 0.001***
Personal pronouns	0.12	0.06, 0.19	< 0.001***
Auxiliary verbs	0.12	0.05, 0.18	< 0.001***
Negation words	0.12	0.05, 0.18	< 0.001***
Cognitive processes	0.12	0.05, 0.18	< 0.001***
Differ	0.12	0.06, 0.18	< 0.001***
Achievement	-0.12	-0.19, -0.06	< 0.001***
Work	-0.12	-0.18, -0.06	< 0.001***
Informal	0.12	0.05, 0.18	< 0.001***
Function words	0.11	0.05, 0.17	0.001**
We (1st person plural)	-0.11	-0.17, -0.05	0.001**
They (3rd person singular)	0.11	0.05, 0.18	< 0.001***
Article	-0.11	-0.17, -0.05	0.001**
Discrepancy	0.11	0.05, 0.17	0.001**
Relative	-0.11	-0.17, -0.04	0.001**
Swear	0.11	0.05, 0.17	0.001**
Tone	-0.1	-0.17, -0.04	0.002**
Six letter words	-0.1	-0.16, -0.04	0.002**
Cause	0.1	0.04, 0.17	0.001**
Feel	0.1	0.04, 0.17	0.001**
Body	0.1	0.04, 0.16	0.002**
Health	0.1	0.04, 0.16	0.002**
Death	0.1	0.04, 0.17	0.001**
Clout	-0.09	-0.15, -0.02	0.008**
Drives	-0.09	-0.16, -0.03	0.004**
Reward	-0.09	-0.15, -0.03	0.006**
Dictionary words	0.08	0.01, 0.14	0.02*
You (2nd person singular)	0.08	0.01, 0.14	0.02*
Number	-0.08	-0.14, -0.01	0.02
Time	-0.08	-0.14, -0.02	0.01*
Internet slang	0.08	0.01, 0.14	0.02*

Prepositions	-0.07	-0.13, 0	0.04*
Positive emotions	-0.07	-0.14, -0.01	0.03*
Female	0.07	0.01, 0.13	0.03*
Tentative	0.07	0, 0.13	0.04*
Power	-0.07	-0.13, -0.01	0.03*
3rd person (she/he, they)	0.06	0, 0.13	0.06
Impersonal pronouns	0.06	0, 0.13	0.06
Insight	0.06	-0.01, 0.12	0.09
Ingest	0.06	-0.01, 0.12	0.08
Leisure	-0.06	-0.13, 0	0.06
Assent	0.06	0, 0.13	0.05
Affect	0.05	-0.02, 0.11	0.15
Friend	0.05	-0.01, 0.12	0.1
Percept	0.05	-0.01, 0.12	0.1
Authentic	0.04	-0.02, 0.10	0.21
Compare	-0.04	-0.11, 0.02	0.21
Interrogative	0.04	-0.02, 0.1	0.21
Quantifier	0.04	-0.02, 0.11	0.21
Affiliation	-0.04	-0.1, 0.02	0.23
Non-fluency words	0.04	-0.02, 0.11	0.19
Period	-0.04	-0.10, 0.03	0.27
Adjective	-0.03	-0.1, 0.03	0.31
Social	0.03	-0.03, 0.1	0.3
Risk	0.03	-0.04, 0.09	0.42
Focus on past	0.03	-0.03, 0.09	0.37
Focus on future	0.03	-0.03, 0.09	0.36
Motion	-0.03	-0.09, 0.04	0.37
Filler	0.03	-0.03, 0.09	0.35
All punctuation	-0.03	-0.09, 0.03	0.36
She/he (3rd person singular)	0.02	-0.04, 0.09	0.45
Male	-0.02	-0.08, 0.05	0.58
Home	0.02	-0.05, 0.08	0.6
Exclamation point	0.02	-0.05, 0.08	0.6
Family	0.01	-0.05, 0.08	0.66
See	-0.01	-0.07, 0.06	0.86
Hear	0.01	-0.05, 0.08	0.69
Religion	-0.01	-0.07, 0.06	0.79
Question mark	0.01	-0.05, 0.08	0.66
Certain	0	-0.07, 0.06	0.95
Money	0	-0.06, 0.07	0.94
Apostrophe	0	-0.06, 0.07	0.94

Bivariate correlations between 87 LIWC text features and current depression severity with 95% confidence intervals (CIs). Approximately 59% of text features are correlated with depression severity at the alpha = 0.05 level. Unadjusted two-sided Pearson correlation with 95% CIs. Source data are provided as a Source Data file.
