# Supplementary material for: Using language in social media posts to study the network dynamics of depression longitudinally
Source: Nat Commun. 2022 Feb 15;13:870. doi: 10.1038/s41467-022-28513-3 (PMC8847554; doi:10.1038/s41467-022-28513-3)
Supplement: Supplementary file 3 — Reporting Summary [file 41467_2022_28513_MOESM3_ESM.pdf]

nature research

Corresponding author(s): Sean W. Kelley, Claire M. Gillan

Last updated by author(s): Nov 30, 2021

Reporting Summary

Nature Research wishes to improve the reproducibility of the work that we publish. This form provides structure for consistency and transparency in reporting. For further information on Nature Research policies, see our Editorial Policies and the Editorial Policy Checklist.

Statistics

For all statistical analyses, confirm that the following items are present in the figure legend, table legend, main text, or Methods section.

n/a	Confirmed	
☐	☒	The exact sample size (n) for each experimental group/condition, given as a discrete number and unit of measurement
☐	☒	A statement on whether measurements were taken from distinct samples or whether the same sample was measured repeatedly
☐	☒	The statistical test(s) used AND whether they are one- or two-sided<br>*Only common tests should be described solely by name; describe more complex techniques in the Methods section.*
☐	☒	A description of all covariates tested
☐	☒	A description of any assumptions or corrections, such as tests of normality and adjustment for multiple comparisons
☐	☒	A full description of the statistical parameters including central tendency (e.g. means) or other basic estimates (e.g. regression coefficient) AND variation (e.g. standard deviation) or associated estimates of uncertainty (e.g. confidence intervals)
☐	☒	For null hypothesis testing, the test statistic (e.g. F, t, r) with confidence intervals, effect sizes, degrees of freedom and P value noted<br>*Give P values as exact values whenever suitable.*
☒	☐	For Bayesian analysis, information on the choice of priors and Markov chain Monte Carlo settings
☒	☐	For hierarchical and complex designs, identification of the appropriate level for tests and full reporting of outcomes
☐	☒	Estimates of effect sizes (e.g. Cohen's d, Pearson's r), indicating how they were calculated

Our web collection on statistics for biologists contains articles on many of the points above.

Software and code

Policy information about availability of computer code

Data collection	Tweets were collected using a data collection app written in Python using the Twitter developer's Application Programming Interface.
Data analysis	Twitter text was cleaned and aggregated by day using a custom Python script. Text analysis was then preformed using the commercially available Linguistic Inquiry and Word Count (LIWC2015). Personalised networks were estimated for each participant using the graphicalVar (version 0.2.4) package, network stability was calculated with the bootnet (version 1.5) package, and mean personalised networks were visualised using the qgraph package (version 1.6.9) in R (3.6.1). Between and within-subjects regression were preformed using the glm (version 3.6.1) and lmer packages (version 3.1-3) also in R (3.6.1).

For manuscripts utilizing custom algorithms or software that are central to the research but not yet described in published literature, software must be made available to editors and reviewers. We strongly encourage code deposition in a community repository (e.g. GitHub). See the Nature Research guidelines for submitting code & software for further information.

Data

Policy information about availability of data

All manuscripts must include a data availability statement. This statement should provide the following information, where applicable:

- Accession codes, unique identifiers, or web links for publicly available datasets
- A list of figures that have associated raw data
- A description of any restrictions on data availability

The raw datasets used and analysed during the current study have sensitive and personally identifiable information. Consequently, only processed and aggregated data are available from the corresponding author on reasonable request.

Field-specific reporting

Please select the one below that is the best fit for your research. If you are not sure, read the appropriate sections before making your selection.

☐ Life sciences ☒ Behavioural & social sciences ☐ Ecological, evolutionary & environmental sciences

For a reference copy of the document with all sections, see nature.com/documents/nr-reporting-summary-flat.pdf

Behavioural & social sciences study design

All studies must disclose on these points even when the disclosure is negative.

Study description	The study involved quantitative cross-sectional associations between personalised network connectivity and current depression severity. Among participants with a depressive episode in the past year, we compared network connectivity within-subject during and outside a depressive episode.
Research sample	Twitter users over 18 years old were recruited primarily from English speaking countries, i.e. U.S., U.K., Ireland, Australia, and Canada, with 86.5% of participants coming from the US or UK. Participants had a mean age of 29.6 years (SD: 10.6) and a majority was female (65.2%). The majority (78.2%) of the sample had completed at least some university level education. The sample is not representative of the general population but is largely representative of the population of Twitter users who tend to be younger and more educated than the general population. The sample was chosen to acquire participants with relatively frequent Twitter use and who were willing to complete self-reported mental health questionnaires.
Sampling strategy	We used a convenience sampling strategy by recruiting either paid users or volunteers targeted through Twitter. We aimed to acquire approximately 1,000 participants in order to have 80% power to detect a correlation of r = 0.09 with alpha = 0.05.
Data collection	Participants were both recruited and tested remotely. Participants were asked to complete a self-report questionnaire remotely and provide their Twitter handle which was used to collect the most recent (max 3,200) tweets and (max 3,200) likes from their account. In the first wave of recruitment, 263 participants completed the Centers for Epidemiologic Studies Depression scale (CES-D 8). In subsequent recruitment waves, the remaining 1,450 participants completed the Zung Self-Rating Depression Scale (SDS) instead. The researchers were not blinded to the study hypothesis.
Timing	Data collection began in March 2019 and ended in April 2020.
Data exclusions	Participants were included for analysis if they were at least 18 years old and had a Twitter account with at least 30 days of tweets and if at least 50% of their tweets were in English. They were also required to pass an attention check, a combination of a captcha and an item with an obvious correct response ("Please select 'A little' if you are paying attention"). Of the 1,713 participants recruited, 99 were excluded due to failing the attention check and a further 668 participants were excluded for either not having at least 30 days of tweets or fewer than 50% of their tweets were in English.
Non-participation	No participants dropped out or declined participation.
Randomization	Participants were not randomly allocated into groups. We observed an association between the length of the text feature time-series, i.e., number of days with Tweets, and network connectivity, and subsequently controlled for the number of days as a sensitivity analysis.

Reporting for specific materials, systems and methods

We require information from authors about some types of materials, experimental systems and methods used in many studies. Here, indicate whether each material, system or method listed is relevant to your study. If you are not sure if a list item applies to your research, read the appropriate section before selecting a response.

Materials & experimental systems

n/a	Involved in the study	
☒	☐	Antibodies
☒	☐	Eukaryotic cell lines
☒	☐	Palaeontology and archaeology
☒	☐	Animals and other organisms
☐	☒	Human research participants
☒	☐	Clinical data
☒	☐	Dual use research of concern

Methods

n/a	Involved in the study	
☒	☐	ChIP-seq
☒	☐	Flow cytometry
☒	☐	MRI-based neuroimaging

Human research participants

Policy information about studies involving human research participants

Population characteristics	Participants had a mean age of 29.6 years (SD: 10.6, range: 18-66), a majority were female (65.2%), currently unemployed (51.6%), and resided in either the U.K. (35.9%) or U.S. (50.7%). More than half (59.0%) of the sample reported at least one depressive episode in the past year (mean: 1.56 episodes, SD: 0.81) with an average duration of 104.06 days (SD: 97.06) and 45.7% reported being diagnosed by a physician with depression at some point in their life.
Recruitment	We recruited 1,713 participants for this study. The majority were recruited on Clickworker (N = 1,395), an online worker platform, and were paid €2.5 for their participation. A smaller number participated voluntarily (i.e. without payment) and were recruited through general advertising on Twitter and in print media (N = 318). Participants who participated voluntarily had significantly higher levels of Twitter engagement, e.g. more Tweets, than those paid for participation. Participants in this study could have self-selected based on their interest in mental health and may have been more likely to consequently tweet about mental health related content than the overall Twitter population. Consequently, we may have observed a rate of depression than is greater than expected, which could lead to an overestimate of our effect size.
Ethics oversight	Approved was granted by the Trinity College Dublin Department of Psychology Research Ethics Committee (Approval ID: SPREC112018-32).

Note that full information on the approval of the study protocol must also be provided in the manuscript.
